# Supplementary material for: ER Stress Induces Cell Cycle Arrest at the G2/M Phase Through eIF2α Phosphorylation and GADD45α
Source: Int J Mol Sci. 2019 Dec 13;20(24):6309. doi: 10.3390/ijms20246309 (PMC6940793; doi:10.3390/ijms20246309)
Supplement: Supplementary file 1 [file ijms-20-06309-s001.pdf]

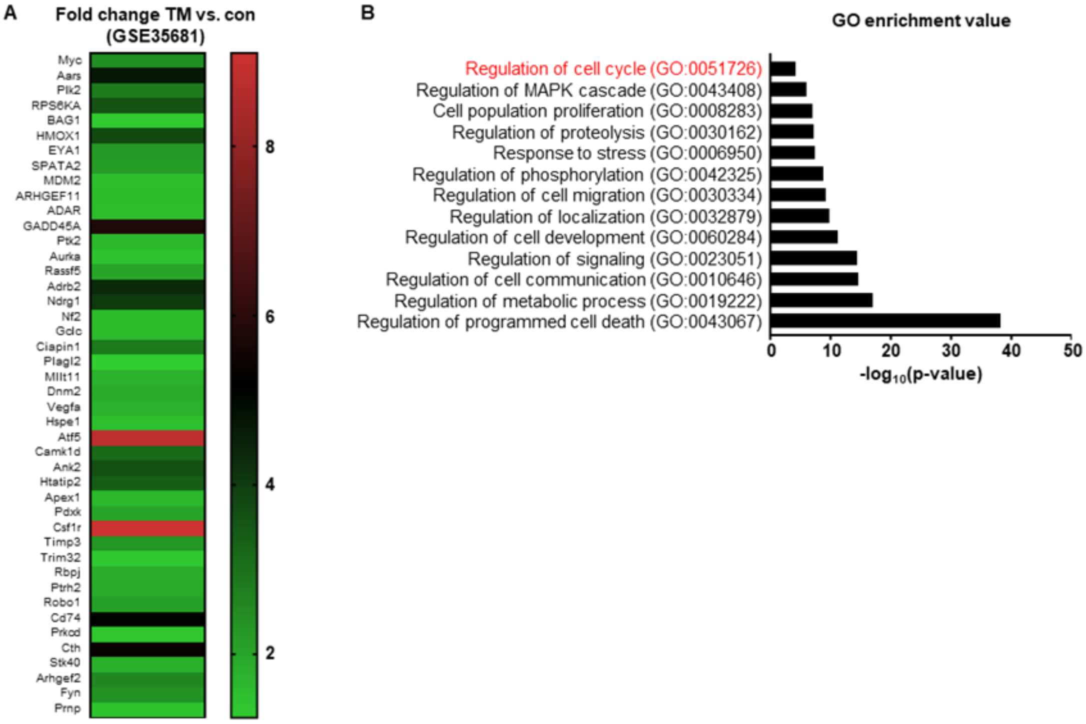

Figure S1. (**A**) Heatmap of the upregulated genes in wild type MEFs with tunicamycin (TM) compared to the control. Data were extracted from GSE35681. (**B**) Gene ontology (GO) analysis using the PANTHER bioinformatics database derived from GSE35681.
